# A Peak Traffic Congestion Prediction Method Based on Bus Driving Time

**DOI:** 10.3390/e21070709

**Published:** 2019-07-19

**Authors:** Zhao Huang, Jizhe Xia, Fan Li, Zhen Li, Qingquan Li

**Affiliations:** 1 Shenzhen Key Laboratory of Spatial Smart Sensing and Services, Shenzhen University, Shenzhen 518060, China; 2 College of Information Engineering, Shenzhen University, Shenzhen 518060, China; 3 College of Computer Science and Software Engineering, Shenzhen University, Shenzhen 518060, China

**Keywords:** intelligent transportation systems, LSTM, road congestion prediction, GPS trajectory, driving time

## Abstract

Road traffic congestion has a large impact on travel. The accurate prediction of traffic congestion has become a hot topic in intelligent transportation systems (ITS). Recently, a variety of traffic congestion prediction methods have been proposed. However, most approaches focus on floating car data, and the prediction accuracy is often unstable due to large fluctuations in floating speed. Targeting these challenges, we propose a method of traffic congestion prediction based on bus driving time (TCP-DT) using long short-term memory (LSTM) technology. Firstly, we collected a total of 66,228 bus driving records from 50 buses for 66 working days in Guangzhou, China. Secondly, the actual and standard bus driving times were calculated by processing the buses’ GPS trajectories and bus station data. Congestion time is defined as the interval between actual and standard driving time. Thirdly, congestion time prediction based on LSTM (T-LSTM) was adopted to predict future bus congestion times. Finally, the congestion index and classification (CI-C) model was used to calculate the congestion indices and classify the level of congestion into five categories according to three classification methods. Our experimental results show that the T-LSTM model can effectively predict the congestion time of six road sections at different time periods, and the average mean absolute percentage error ($\bar{MAPE}$) and root mean square error ($\bar{RMSE}$) of prediction are 11.25% and 14.91 in the morning peak, and 12.3% and 14.57 in the evening peak, respectively. The TCP-DT method can effectively predict traffic congestion status and provide a driving route with the least congestion time for vehicles.

## 1. Introduction

With the rapid growth of urban vehicles, traffic congestion has become more serious, which not only impacts people’s travel but also restricts the stable development of the urban economy [1,2,3]. According to statistics, the most influential Chinese cities suffer a daily economic loss of $1 billion due to traffic congestion [4]. Therefore, traffic congestion has become one of the most urgent issues in modern cities, especially recurrent congestion such as peak periods in the morning and evening. Due to of the complexity of roads and the instability of traffic flow, it is a great challenge to obtain current or future road traffic conditions in different road segments. Targeting the challenge, a variety of traffic congestion prediction methods have been proposed [5,6,7,8,9,10]. However, it is still a great challenge to accurately and steadily reflect road traffic conditions due to large fluctuations of traffic flow and floating car speeds.

In this paper, we propose a method of traffic congestion prediction based on bus driving time (TCP-DT) to improve prediction accuracy during the peak periods of the working day. We chose the driving time of buses between two bus stations to predict the traffic congestion level of the road section, because the speed of buses is relatively stable compared with the floating speed of cars, and their driving regularity is strong. The period from 11:00 to 13:00 in the off-peak period on a sunny day is considered to be the non-congestion driving time. In the TCP-DT, (1) a map-matching method is adopted to match the bus location with the bus station location, and then the driving times of different road segments in the morning and evening peak periods are extracted; (2) the weather and labels of congestion times are converted into one-hot code, which is aggregated into input data with the historical congestion times, and the congestion time prediction based on the long short-term memory (T-LSTM) model is used to train and predict future congestion times [11,12,13]; and (3) the congestion index and classification (CI-C) model is adopted to calculate the traffic congestion index of different road sections by using the predicted congestion time. Finally, we evaluate the performance of the classification methods based on information entropy [14,15].

The main contributions of this paper include the following: (1) The TCP-DT is used to calculate the vehicle congestion indices by classifying the driving times of buses on the designated road sections during the morning and evening peak periods. (2) Six road segments, 50 buses, 66 working days, and 66,228 bus driving time records are used to provide congestion evaluation and prediction in Guangzhou, China.

The rest of the paper is structured as follows. Section 2 reviews related work regarding urban traffic congestion prediction. The proposed method of congestion prediction, including the framework, the T-LSTM model, and the CI-C model, are described in Section 3. Section 4 shows the experiment results. Finally, the conclusion and analysis are presented in Section 5.

## 2. Literature Review

In this section, an overview of traffic congestion prediction methods is presented. Yang [16] regarded traffic congestion prediction as a binary classification problem, and comparable accuracy is retained after reducing the dimensionality of input data by feature selection methods. The JamBayes model with temporal traffic variables was proposed to predict traffic congestion by Horvitz et al. [17]. Kim and Wang [18] provided an analytic framework to predict traffic congestion based on Bayesian network. A naive Bayes (NB) classifier model was proposed by Wang and Kim [19] to predict urban congestion and traffic incidents using actual incidents and weather data. Gajewski and Rilett [20] estimated link travelling time correlation and looked for heavier traffic congestion by a Bayesian-based approach. Zhou et al. [21] proposed a method based on a least squares support vector machine (LS-SVM) classification and a regression model to predict traffic conditions using floating car data. Ando et al. [22] proposed a method to predict short-term traffic congestion through a pheromone mechanism and a simulation based on real-world traffic data is used to evaluate the method performance. Han and Shi [23] provided an online prediction method based on Random Forest (RF) to predict traffic congestions by using the real-time data. Mishra et al. [24] proposed a traffic congestion prediction model based on multiple symbol Hidden Markov Model by considering the contribution of each congestion causing or reflecting factors, which could adapt to the road network. Kong et al. [25] proposed a novel approach to estimate and predict the urban traffic congestion using floating car trajectory data. Floating cars is used to probe urban real-time traffic flows, which is converted to congestion state by a congestion state fuzzy division module. Gilmore and Abe [26] described an ATMS blackboard architecture; the architecture includes the Hopfield neural network model and a backpropagation model, which is used to control traffic signal light and predict urban traffic congestion respectively.

Shi et al. [27] proposed an adaptive cubic surface traffic flow model considering time-varying and space-varying information [28] to predict urban traffic status. An approach based on the multivariate spatiotemporal autoregressive model was provided by Min and Wynter [29] to predict real-time traffic conditions and find the regularity of traffic congestion changing with traffic flow. A hybrid learning framework was provided to combine estimation results of freeway traffic density status from multiple macroscopic traffic flow models by Li et al. [30]. Xu et al. [31] predicted traffic flow by a spatiotemporal variable selection method based on a support vector regression (SVR) model. Tseng et al. [32] proposed a SVM-based real-time highway traffic congestion prediction (SRHTCP) model to collect road data and used fuzzy theory to evaluate the real-time traffic level considering road speed, road density, road traffic volume, and rainfall on road sections.

Yoon et al. [33] estimated traffic conditions by using spatial and temporal speed information. Kong et al. [34] presented a method based on a curve-fitting and vehicle-tracking mechanism to predict traffic states through the spatiotemporal average velocity extracted from vehicles’ GPS. Jia et al. [35] provided a smart traffic prediction system, which used the sliding windows to process real-time data stream and make regression analysis by autoregressive integrated moving average model (SWARIMA) to predict congestion trends considering speed, time, and location information. Feng et al. [36] used GPS probe data to estimate arterial travel time states.

Helbing et al. [37] speculated on conditions and features of traffic congestion status and provided empirical evidence to prove the existence of traffic state phases. Cohn introduced the TomTom congestion index to identify areas of concern and alleviate bottlenecks [38], and to create an objective benchmark to evaluate congestion levels [39].

The major limitation in the above-mentioned studies is that the GPS data of floating cars usually have unstable speed and weak regularity, which often results in low prediction accuracy. Targeting these issues, this paper introduces a peak traffic congestion prediction method based on bus driving time to improve the prediction accuracy for morning and evening peak periods.

## 3. TCP-DT Method

In this section, we elaborate a traffic congestion prediction method based on bus driving time to alleviate traffic pressure, which can predict future driving time by the T-LSTM model, and adopt three classification methods to classify congestion levels of a specific road section by the TomTom congestion index.

### 3.1. Framework

**Figure 1:**
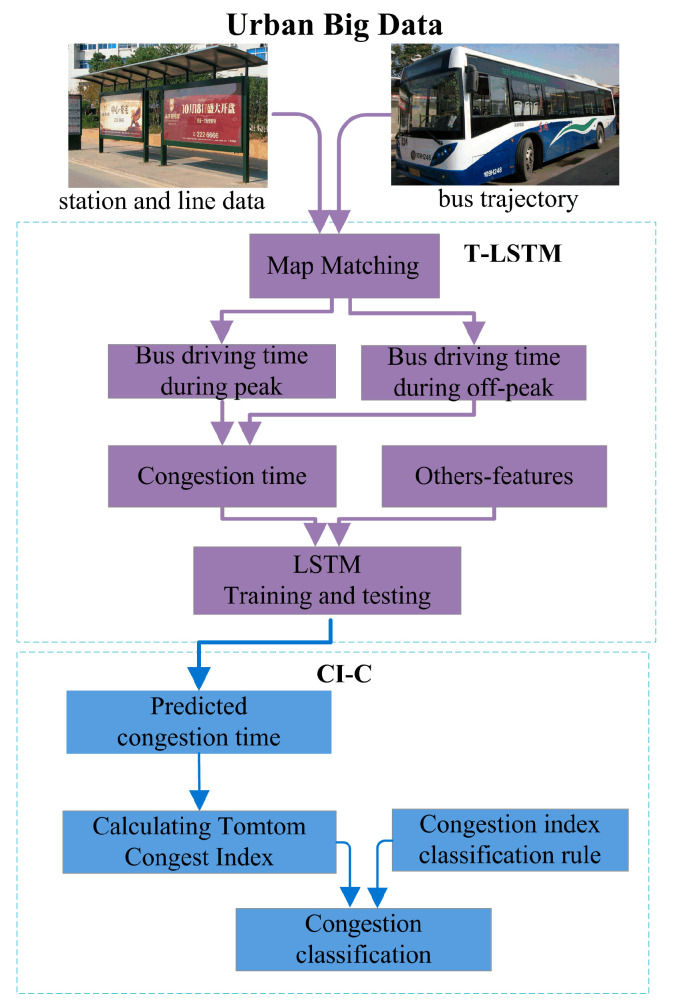
Framework of the traffic congestion prediction based on bus driving time (TCP-DT) method. LSTM, long short-term memory; CI-C, congestion index and classification.

The TCP-DT method consists of two components, the T-LSTM prediction model and the CI-C model, as shown in Figure 1.

The first component mainly matches the bus trajectory data to the bus lines and obtains the arrival and departure times at different stations. Then, LSTM is used to predict future congestion times of buses in specific road sections during morning and evening peaks by combining weather and historical congestion times. The second component calculates the congestion index and classifies it into five categories using methods of geometric interval, equal interval, and natural break. Then, the information entropy of the three methods is calculated separately. Finally, the characteristics of the three classification methods are compared and discussed.

### 3.2. T-LSTM Model

#### 3.2.1. Driving Time Speculation

**Figure 2:**
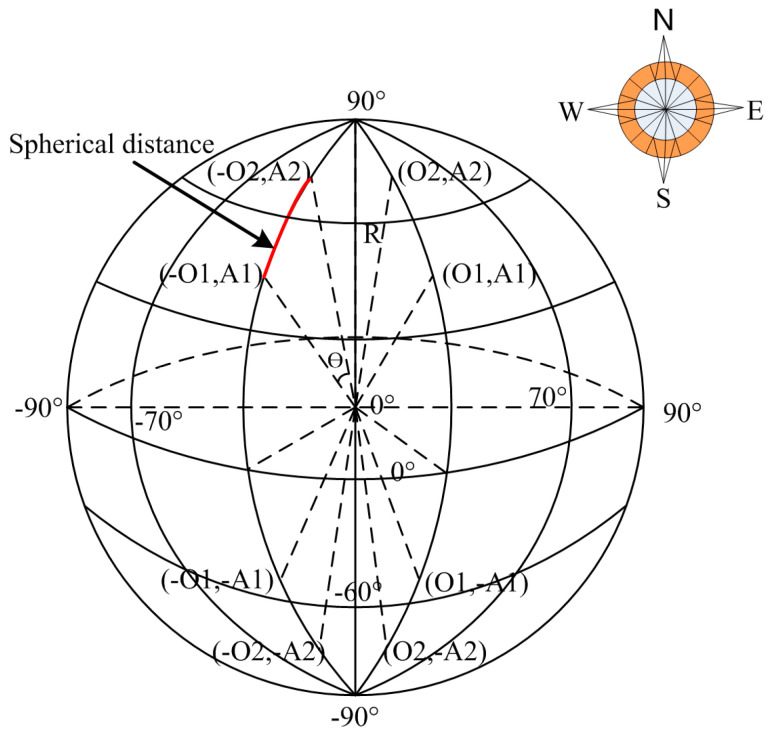
Longitudinal division based on the benchmark of 0° longitude.

The purpose of this section is to obtain the driving times of buses from one station to another. The instantaneous locations of buses should be matched to the locations of bus lines according to the trajectory of the bus. The original GPS trajectory points of buses are low-frequency sampled. This paper adopts a map-matching method for low-frequency floating buses to restore their space-time trajectory [40]. Then, the bus trajectory is interpolated in a uniform speed space in seconds by the average speed interpolation algorithm. Finally, the nearest neighbor rule of spherical distance is adopted to match the arrival and departure times from the bus station. The spherical distance between the instantaneous location of a bus and the location of a bus station can be calculated by the following equations, as shown in Figure 2.

To adjust the longitude and latitude range from 0° to 180°, first, assume that the latitude and longitude of 2 points are (*O*_1_, *A*_1_) and (*O*_2_, *A*_2_), respectively. According to the benchmark of 0° longitude:

if $O_{1}$ is east longitude:
  (1) $$MO_{1}=O_{1}$$
if $O_{1}$ is west longitude:
  (2) $$MO_{1}=-O_{1}$$
if $A_{1}$ is north latitude:
  (3) $$MA_{1}=90-A_{1}$$
if $A_{1}$ is south latitude:
  (4) $$MA_{1}=90+A_{1}$$

Then, the converted latitude and longitude are indicated as (*MO*_1_, *MA*_1_) and (*MO*_2_, *MA*_2_), respectively, and the distance between 2 points can be obtained using trigonometric derivation by Equations (5) and (6):

(5) $$C=\sin\left(MO_{1}\right)\times \sin\left(MO_{2}\right)\times \sin\left(MA_{1}-MA_{2}\right)+\cos\left(MO_{1}\right)\times \cos(MO_{2})$$

(6) D=R×arccos(C)×π÷180

In Equation (6), R represents the radius of the earth. Then, the arrival and departure times are captured.

**Figure 3:**
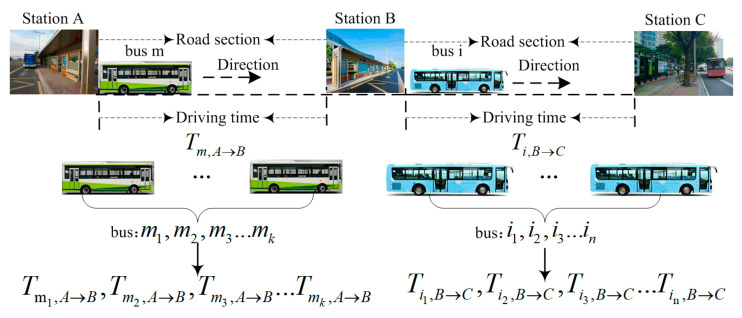
Bus driving time diagram.

Figure 3 shows the process of buses driving from one station to another. Driving time is defined as the interval between departure time at one station and arrival time at the other station. The time intervals of bus $m_{1},m_{2},m_{3}\ldots m_{k}$ driving from station A to station B are denoted as $T_{m_{1},A\to B},T_{m_{2},A\to B},T_{m_{3},A\to B}\ldots T_{m_{k},A\to B}$, respectively. Similarly, the time intervals from station B to station C are defined as $T_{i_{1},B\to C},T_{i_{2},B\to C},T_{i_{3},B\to C}\ldots T_{i_{n},B\to C}$. Then, driving time is defined as $T_{i\to j}^{}$, and the calculating equation is as follows:

(7) $$T_{b,i\to j}^{}=T_{b,\;j}-T_{b,i}$$

In this equation, $T_{b,i}$ indicates the departure time of bus b from station i, $T_{b,\;j}$ denotes the arrival time of bus b at station j, and b represents the label of the bus.

#### 3.2.2. Calculating Congestion Time

There are many cars in the city and the speed of buses varies, thus it is difficult to obtain the nonblocking driving times of buses in different road sections. Generally, fewer people are on the roads from 11:00 to 13:00, therefore traffic congestion rarely occurs. In our method, we regard the average driving time of 50 buses during the time range in a specific road section as the standard driving time of this road section without traffic congestion, and the calculation equation is as follows:

(8) $$T_{i\to j}^{s}=\frac{\sum_{b=1}^{N}T_{b,i\to j}^{}}{N}$$

In this equation, $T_{i\to j}^{s}$ indicates the standard driving time from station i to station j, and N denotes the number of buses. The congestion time is the interval between the driving time during peak period and standard driving time, as defined by Equation (9):

(9) $$T_{i\to j}^{con}=T_{i\to j}^{peak}-T_{i\to j}^{s}$$

where the congestion time from station i to j is denoted as $T_{i\to j}^{con}$, and $T_{i\to j}^{peak}$ represents the driving time from station j to i.

#### 3.2.3. Congestion Time Prediction

**Figure 4:**
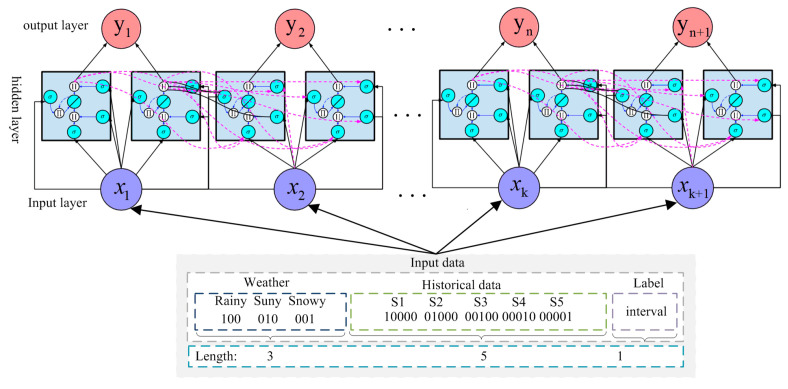
Structure of time prediction based on long short-term memory (T-LSTM) model.

The aim of the T-LSTM model used in this research is to predict future congestion times of buses on specific road sections, and the structure of this model is shown in Figure 4.

The input is composed of weather and historical data. Firstly, the peak period is divided into morning and evening peaks; morning peak is from 07:00 to 09:00, and evening peak is from 17:30 to 19:30. Secondly, the weather is divided into rainy, sunny, and snowy, which is encoded into a 3-bit one-hot code. Thirdly, the historical congestion times of buses in specific sections are divided into five segments, which are converted into 5-bit one-hot codes. The values of congestion times are regarded as labels. Finally, all features are aggregated to form the input data, which has dimensions of 1×8.

**Figure 5:**
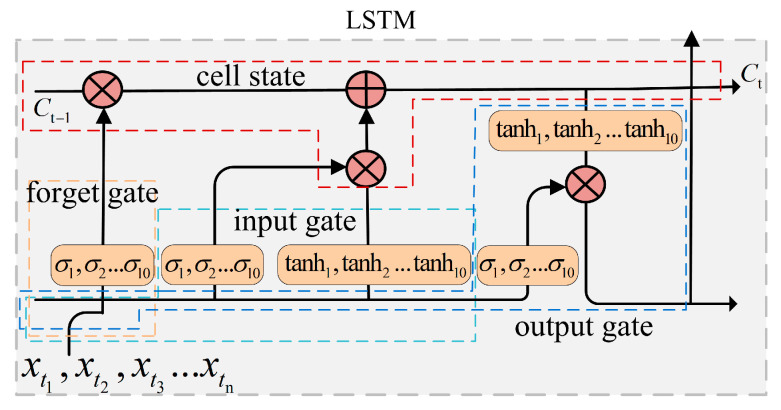
Structure of LSTM Cell.

LTSM is adopted to predict future congestion times due to the advantage of processing and predicting events with long intervals and delays in time series, which generally includes forgetting gate, input gate and output gate and cell state. Figure 5 provides the structure of LSTM cell. (1) The effect of forgetting gate is to control whether the hidden cell state of the front layer is forgotten by a certain probability, which includes ten sigmoid function. (2) The input gate is responsible for processing the input of the current sequence position, which uses sigmoid and tanh activation function. (3) The aim of cell state is to preserve output information from the previous layer and add useful information of the current layer, Then, this information will be transferred to the next layer. (4) The function of the output gate is to output some important information and discard the unnecessary part, which includes ten sigmoid function. LSTM used in our method consists of 3 LSTM cells, and each cell contains 10 neurons. Finally, the k×1 dimension output is obtained after inputting n×8 dimension data.

A number of customizations was conducted on the LSTM framework according to this application. We adopted a four-step tuning process for the LSTM customization: (1) we first set an acceptable target for predicting results (loss value); (2) based on the previous research experience [41], we preliminarily set our parameter values on the basis of predecessor’s prediction parameters and observe the changes of loss. According to the change trends, we preliminarily determine the range of each hyper-parameter; (3) in the process of LSTM adjustment, we adjust one hyper-parameters at a time, and we observe the trend of loss (including train loss and validation loss) change; and (4) during the whole training process, if the change of train loss value and validation loss value showed a stabilized trend, we stop the adjustment and save the value of the hyper-parameter. Otherwise, we constantly adjust the value of the hyper-parameter, iterate and train until loss drops and finally stabilizes.

### 3.3. CI-C Model

#### 3.3.1. Calculating TomTom Congestion Index

In order to reflect the degree of traffic congestion, we introduce the TomTom congestion index (CI) as an indicator to measure congestion levels. This CI reflects the degree of deviation between actual and standard driving times, which is widely used for traffic management in some cities in North America and Europe. The calculation equation is as follows:

(10) $$CI_{i\to j}=\frac{T_{i\to j}^{con}}{T_{i\to j}^{s}}\times 100\%$$

where $CI_{i\to j}$ indicates congestion index from station i to station j.

#### 3.3.2. Classification of Congestion Level

To better reflect the congestion of traffic road sections, we take the average congestion index of each time segment during peak periods to measure the congestion status. Meanwhile, geometric interval, equal interval, and natural break classification methods are used to divide traffic congestion into five grades: better smooth, normal smooth, mild congestion, moderate congestion, and severe congestion. For equal interval classification, the range of the congestion index is divided into five sub-ranges of equal size. Natural breakpoint classification divides the range of the congestion index into five sub-ranges by setting relatively different values as boundaries. Geometric interval classification creates classification intervals based on group spacing with a geometric series to divide the range of the congestion index into five classes.

#### 3.3.3. Calculating Information Entropy

In this paper, to obtain the differences between the three classification methods, information entropy is used to measure the amount of information of the methods. The calculation of information entropy is shown in Equation (11):

(11) $$H_{c}(x)=-\sum_{s=1}^{5}p_{c}(s)\log p_{c}(s)$$

In this equation, $H_{c}(x)$ denotes the information entropy of c, which indicates a method of classification used in our paper, s represents the label of congestion levels, and $p_{c}(s)$ delegates the probability that the congestion level accounts for the proportion of all congestion level quantities. Equation (12) shows the calculation method of the probability:

(12) $$p_{c}(s)=\frac{N_{c,}{}_{s}}{N_{c}}$$

In this expression, $N_{c}$ indicates the number of classification method c, and $N_{c,}{}_{s}$ represents the number of congestion level s in classification method c.

## 4. Experiment Results and Discussion

### 4.1. Data Predescription

The dataset used in our experiment covered 66 working days and 6 road sections from 25 March to 29 June 2015 in Guangzhou, China, containing bus station, line vector, and bus trajectory data. 

#### 4.1.1. Bus Station and Line Vector Data

The open API of Baidu Maps provided access to collect the data, which included station ID, name, latitude and longitude, and line label. The detailed geographic information was extracted from the line vector data, which included nine stations and a total of 66,228 data records; these stations were divided into six road sections, and the origin and destination stations are listed in Table 1.

#### 4.1.2. Bus Trajectory Data

GPS terminal devices are installed on buses to collect trajectory data, and a low-frequency data sampling method was adopted with a sampling frequency of 60 HZ. The bus plate number, time of data acquisition, instantaneous speed, direction, latitude, and longitude were recorded in the bus trajectory information. Detailed descriptions of the data in the dataset are shown in Table 2.

### 4.2. Data Preprocessing

After collecting the buses’ GPS trajectory data, the driving times for six road sections could be deduced by speculating on arrival and departure times, then the congestion times of these road sections could be obtained by comparing them to standard times.

**Figure 6:**
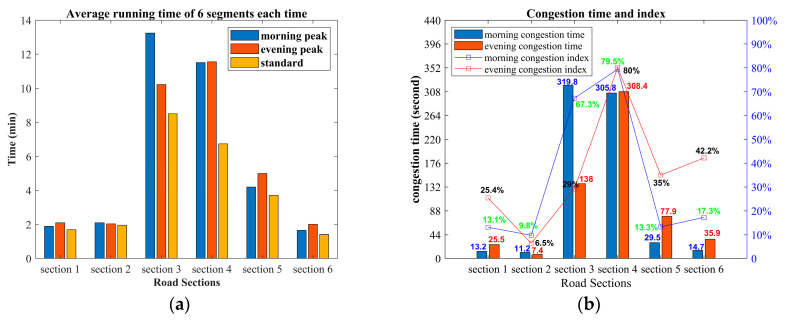
(**a**) Average driving time of six road sections; (**b**) distribution of congestion time and index.

Figure 6a shows the average driving times of buses in the six sections. Blue and orange bars in the charts denote driving times during morning and evening peaks, and yellow bars indicate standard driving times. As we can see from the height of the pillar, the driving times of all road sections in peak periods are always longer than the standard driving times, which explains the occurrence of traffic congestion during peak periods.

Figure 6b illustrates the average congestion times and indices for six road sections during peak periods. The congestion times of road section 2 are 11.2 s and 7.4 s, and the congestion indices are 9.8% and 6.5%, respectively, for the two periods, which are the smallest of the six road sections, thus this road section is relatively smooth. Road section 3 has the longest morning congestion time, 319.8 s, and road section 4 has the longest evening congestion time, 308.4 s. The maximum congestion index of the six road sections is 79.5% and 80.0%, respectively, for the two periods, indicating that the congestion level is severe. In summary, traffic jams during the morning peak period on road section 3 and during the evening peak period on road section 4 are the most serious, and traffic congestion during morning and evening peak periods on road section 1 is the lightest.

### 4.3. Prediction Results

#### 4.3.1. Parameter Descriptions

We used 80% of the dataset to train the prediction model and the remaining 20% to test the performance of the model. The detailed parameters are listed in Table 3.

#### 4.3.2. Performance Indicators

In the process of testing, the mean absolute percentage error ($\bar{MAPE}$) and root mean square error ($\bar{RMSE}$) are adopted as indicators to measure the performance of the prediction model [42]. The calculations of $\bar{MAPE}$ and $\bar{RMSE}$ are shown as Equations (13) and (14), respectively:

(13) $$\bar{MAPE}=\frac{1}{K}\sum_{j=1}^{K}\frac{\left|t_{h}(j)-{\tilde{t}}_{h}(j)\right|}{t_{h}(j)}\times 100\%$$

(14) $$\bar{RMSE}=\sqrt{\frac{\sum_{j=1}^{K}|t_{h}(j)-{\tilde{t}}_{h}(j){|}^{2}}{K}}$$

In these equations, $t_{h}(j)$ denotes real bus driving time inferred from GPS trajectory, and ${\tilde{t}}_{h}(j)$ represents the predicted bus running time using the proposed T-LSTM model.

$\bar{MAPE}$ and $\bar{RMSE}$ are often used to measure the difference between predicted and real values. $\bar{MAPE}$ reflects the percentage of difference and real values, and smaller percentages represent higher prediction accuracy. However, it is not enough to judge the difference only considering $\bar{MAPE}$ when the difference is small. Therefore, $\bar{RMSE}$ is introduced to assist in measuring the difference.

#### 4.3.3. Prediction of Congestion Time

Prediction results of congestion times for six road sections are shown in Table 4, including morning and evening peak periods. During the morning peak period, the lowest and highest $\bar{MAPE}$ are 8.0% and 12.7%, respectively, which indicates that the prediction accuracy in section 3 is higher than in other sections, and the accuracy in section 1 is the worst. Meanwhile, the lowest and highest $\bar{RMSE}$ are 3.05 and 35, respectively, which indicates that the difference in section 6 between prediction and reality is the smallest, and the most obvious difference is in section 3. The average $\bar{MAPE}$ and $\bar{RMSE}$ are 11.25% and 14.91, respectively. During the evening peak period, the lowest and highest $\bar{MAPE}$ are 9.7% and 15%, respectively, which indicates that the prediction result of section 5 is the best, and section 3 is the worst. The lowest and highest $\bar{RMSE}$ are 2.9 and 44.5, respectively, which indicates that the maximum difference is in section 4 and the smallest difference is in section 2. The average $\bar{MAPE}$ is 12.3% and $\bar{RMSE}$ is 14.57 in the evening peak.

**Figure 7:**
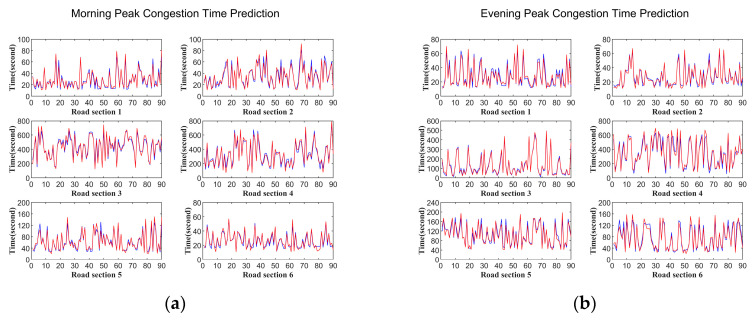
Distribution of real and predicted congestion time: (**a**) morning peak, (**b**) evening peak.

To better illustrate the experimental results, we extracted 90 congestion times for each road section to show the predicted results. Figure 7 depicts the predicted and real congestion times of the six road sections during morning and evening peak periods. The red curve depicts the real congestion times of buses in the six road sections, and the blue curve represents the predicted congestion times. From the picture, we can see that the changing trend of the predicted value curve is very close to the real value curve, which indicates that the predicted curve can reflect the change of real values perfectly.

In summary, the T-LSTM model can accurately and steadily predict the congestion times of morning and evening peak periods to provide information on road status in advance, and lays a foundation for calculating congestion index and classifying congestion levels.

#### 4.3.4. Classification of Congestion Levels

**Figure 8:**
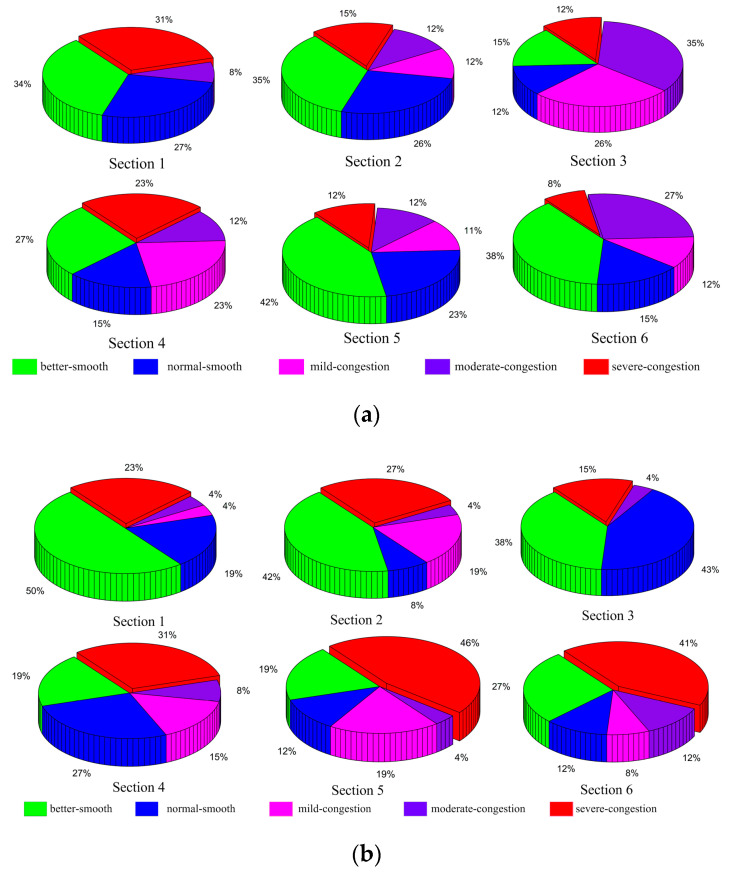
Equal interval classification of predicted data: (**a**) morning peak, (**b**) evening peak.

**Figure 9:**
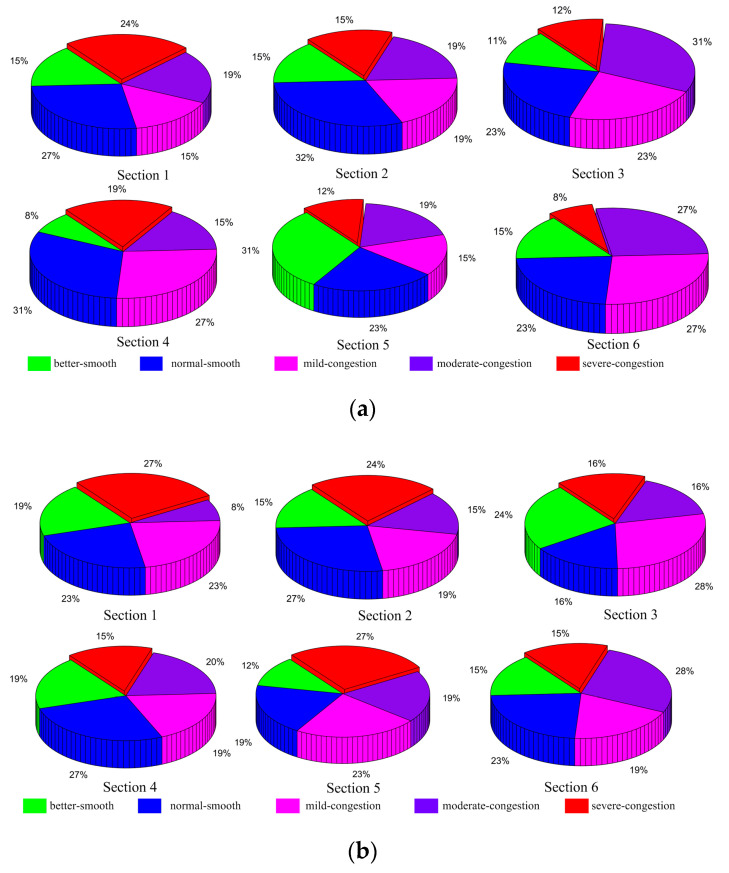
Natural breakpoint classification of predicted data: (**a**) morning peak, (**b**) evening peak.

**Figure 10:**
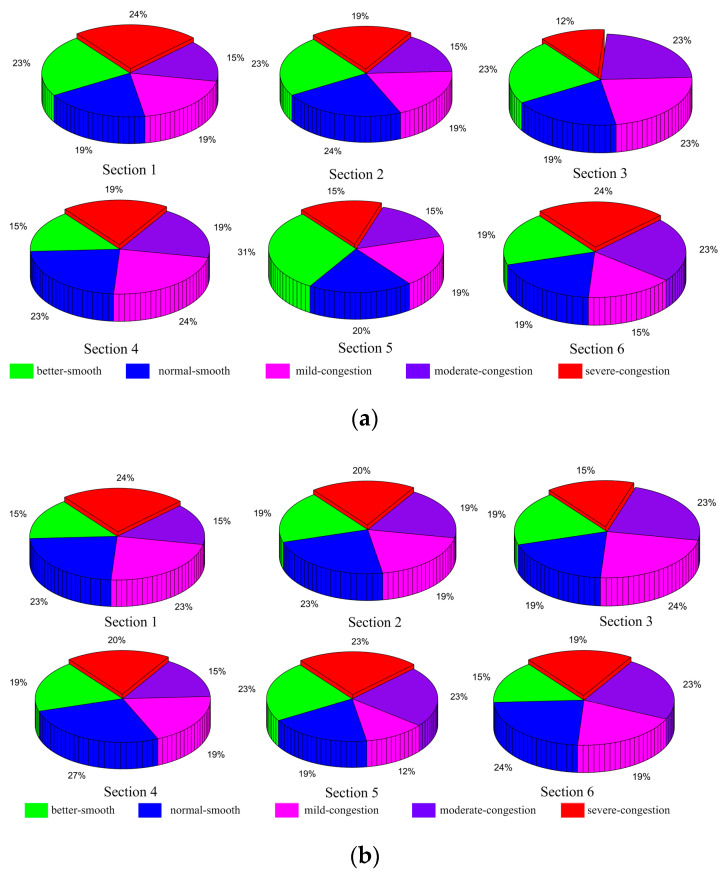
Geometric interval classification of predicted data: (**a**) morning peak, (**b**) evening peak.

There are three steps for classifying congestion. Firstly, the congestion index is calculated using times predicted by the T-LSTM model. Secondly, the average daily congestion indices of morning and evening peaks are calculated. Thirdly, the congestion levels of morning and evening peaks are classified into five grades by the three classification methods. In order to better present the distribution of congestion levels in six sections, the proportion of each grade for the predicted 13 days is obtained, shown in Figure 8, Figure 9 and Figure 10.

Figure 8 shows the proportion of five congestion grades for the six sections during peak periods by the equal interval classification. During the morning peak period, the proportion of better smooth is larger than other grades in road sections 1, 2, 4, 5 and 6, which account for 34%, 35%, 27%, 42%, and 38%, respectively. The proportion of moderate congestion is 35% in road section 3, the largest of all grades. The congestion proportions of the six road sections are 39%, 39%, 73%, 58%, 35%, and 47%. Similar to the morning peak, the proportion of better smooth in sections 1, 2, and 3 during the evening peak period are smaller than the others, which are 50%, 42%, 38%, and severe congestion accounts for 31%, 46%, and 41% in the other sections. The congestion proportions are 31%, 50%, 19%, 54%, 69%, and 61%.

Figure 9 illustrates the proportions by using the natural breakpoint classification method. During the morning peak period, normal smooth accounts for larger proportions in road sections 1, 2, and 4, which are 27%, 32%, and 31%. Moderate congestion accounts for 31% in section 3, and better smooth accounts for 31% in section 5, and both mild and moderate congestion account for 27% in section 6, representing the largest proportions. The congestion proportions of the six sections are 58%, 53%, 66%, 61%, 46%, and 62%. During the evening peak period, the proportion of normal smooth of both sections 2 and 4 is 27%. Similarly, the proportion of severe congestion in both sections 1 and 5 is also 27%, and mild congestion and moderate congestion both account for 28% in sections 3 and 6. The proportions of congestion in the evening peak are 58%, 58%, 60%, 54%, 69%, and 62%.

Figure 10 shows the result of geometric interval classification. The highest proportions are 24%, 24%, 24%, 31%, and 24% for sections 1, 2, 4, 5, and 6, respectively, indicating severe congestion, normal smooth, mild congestion, better smooth, and severe congestion. Better smooth, mild congestion, and moderate congestion each account for 23% in section 3. The proportions of congestion are 58%, 53%, 58%, 62%, 49%, and 62%. The largest proportions of these sections are 24%, 23%, 24%, 27%, 23%, and 24%, and the congestion proportions are 62%, 58%, 62%, 54%, 58%, and 61%.

The congestion proportions of six road sections using three classification methods are summarized in Table 5.

In summary, comparing the three classification methods, we can conclude that the geometric interval classification method has the most uniform distribution and the equal interval classification method has the worst distribution.

#### 4.3.5. Calculating Information Entropy

The results of the three classification methods in the previous section may not fully reflect the magnitude of information. Therefore, the information entropy of the six sections by using the three classification methods is calculated separately, as shown in Table 6. All the information entropy by geometric interval classification is larger than with the other methods, and there is a big difference compared with the equal interval method and a small difference compared with the natural breakpoint method.

Table 7 shows the total information entropy of the three classification methods during the morning and evening. From the table, the information entropy of the geometric interval method is larger than the others, and the morning and evening information entropy is the largest. Conversely, the equal interval method is the smallest for morning and evening information entropy, and the natural breakpoint method has moderate information entropy.

To sum up, there are large differences in the classification results of the same data when comparing the information entropy of the three classification methods, especially between equal interval and geometric interval, and geometric has the largest information entropy in all sections. Therefore, geometric interval performs better than the others in terms of information entropy.

### 4.4. Discussion

Based on the experimental results, the geometric interval method displayed more road status information (larger information entropy) and a more balanced congestion distribution (Figure 6, Figure 7 and Figure 8). In other words, geometric interval classification generally outperformed equal interval and natural breakpoint classification in terms of information entropy and distribution. However, the disadvantage of the geometric interval method is that congestion grades usually cannot be divided according to historical experience and the difference of each grade is maximized inconspicuously. Meanwhile, the natural breakpoint method maximizes the difference of each grade, but the limitation is finding the grade with the smallest variance by computing the variance of each grade, and the amount of computation is enormous. Therefore, there is a trade-off between the geometric interval and natural breakpoint methods.

In the future, we intend to extend our peak congestion prediction method by considering more factors. Then, we plan to utilize our approach to optimize shortest-time planning for a variety of transportation activities [43] in Guangzhou City, China, and to consider multiple factors and increase the applicability of this method in our future work.

## 5. Conclusions

In this paper, a peak traffic congestion prediction method based on bus driving time was used to predict the peak traffic congestion in large-scale urban areas. A map-matching method was adopted to match the bus trajectory data and bus sample points. Then, the bus driving time in different road sections during peak periods was extracted, and an LSTM neural network was used to predict the traffic congestion time. In order to improve the stability and reliability of prediction, the weather was also taken into consideration. Our method extracts the driving time of different road sections to measure the state of traffic congestion and divide the state of traffic into five grades using three classification methods. By using data of 66 working days for six road sections and a total of 66,228 bus driving records in Guangzhou City, our experimental results show that the average $\bar{MAPE}$ of morning and evening peaks is 11.25% and 12.3%, and the average $\bar{RMSE}$ of morning and evening peaks is 14.91 and 14.57, respectively. However, the limitation of our current approach is that the congestion prediction of the dedicated bus lane sections is invalidated. In future, we will combine bus and floating car data to overcome the challenges posed by the dedicated bus lane.

## Figures and Tables

**Table 1:** Road sections used in the experiment.

Origin	Destination	Section Label
Luoshou south residence	Shangjiao	section 1
Shangjiao	Wuzhou decoration city	section 2
Wuzhou decoration city	Longtan village	section 3
Datang village	Tianhe south	section 4
Tianhe south	Tianhe bus station	section 5
Chuangde shoe factory	West second village	section 6

**Table 2:** Summary of experimental data.

Data Type	Description	Feature
Station and line data	Six road sections, total of 66,228 daily records, covering 66 working days	Station name and bus line, ID, latitude, and longitude
Bus trajectory data	Low-frequency sampling every 60 s	Direction angle, time of data acquisition, bus plate number, instantaneous latitude, longitude, and speed

**Table 3:** Detailed parameter settings of LSTM model.

Parameter	Description	Value
rnn_unit	Number of hidden layer neurons	10
lstm_layers	Number of hidden layers	3
learning_rate	Learning rate in training process	0.0006
keep_prob	Probability of retained neurons in dropout layer	0.5
batch_size	Size of batch training	40
time_step	Time step	30

**Table 4:** Summary of prediction results.

Station	Peak	$\bar{MAPE}$	$\bar{RMSE}$
section 1	Morning	12.7%	4.02
	Evening	13.5%	3.84
section 2	Morning	11.5%	4.70
	Evening	11.3%	2.90
section 3	Morning	8.0%	35.00
	Evening	15%	13.67
section 4	Morning	12.6%	34.20
	Evening	12.1%	44.50
section 5	Morning	10.8%	8.50
	Evening	9.7%	11.50
section 6	Morning	11.9%	3.05
	Evening	12.3%	11.06

**Table 5:** Proportions of congestion.

Method	Peak	Section 1	Section 2	Section 3	Section 4	Section 5	Section 6
Equal Interval	Morning	39%	39%	73%	58%	35%	47%
	Evening	31%	50%	19%	54%	69%	61%
Natural Breakpoint	Morning	58%	53%	66%	61%	46%	62%
	Evening	58%	58%	60%	54%	69%	62%
Geometric Interval	Morning	58%	53%	58%	62%	49%	62%
	Evening	62%	58%	62%	54%	58%	61%

**Table 6:** Information entropy of six road sections.

Method	Peak	Section 1	Section 2	Section 3	Section 4	Section 5	Section 6
Equal Interval	Morning	1.85	2.18	2.18	2.26	2.10	2.11
	Evening	1.81	1.97	1.65	2.19	1.98	2.06
Natural Breakpoint	Morning	2.28	2.26	2.22	2.19	2.24	2.21
	Evening	2.23	2.28	2.28	2.30	2.28	2.28
Geometric Interval	Morning	2.30	2.30	2.29	2.30	2.26	2.30
	Evening	2.29	2.32	2.30	2.30	2.29	2.30

**Table 7:** Total information entropy of three classification methods.

Peak	Equal Interval	Natural Breakpoint	Geometric Interval
Morning	12.68	13.40	13.76
Evening	11.67	13.64	13.79
Total	24.35	27.04	27.55
